# Self-Care Practices and Perspectives in Managing Coronary Heart Disease Patients: A Qualitative Study

**DOI:** 10.3390/nursrep14040237

**Published:** 2024-10-31

**Authors:** Setyowati Setyowati, Aria Wahyuni, Hananto Adriantoro, Kasiyah Junus, Eric Umar, Nelsensius Klau Fauk, Hidayat Arifin

**Affiliations:** 1 Faculty of Nursing, Universitas Indonesia, Depok 16424, West Java, Indonesia; 2 Departement of Nursing, Faculty of Health, Muhammadiyah University of West Sumatera, Bukittinggi 26181, West Sumatera, Indonesia; 3 Department of Cardiology and Vascular Medicine, Faculty of Medicine, Universitas Indonesia, Central Jakarta 10430, Jakarta, Indonesia; hantoro.0411@gmail.com; 4 Harapan Kita National Cardiovascular Center, West Jakarta 11420, Jakarta, Indonesia; 5 Faculty of Computer Science, Universitas Indonesia, Depok 16424, West Java, Indonesia; kasiyah@cs.ui.ac.id; 6 Department of Health Systems and Policy, Kamuzu University of Health Sciences, Blantyre 52X8+782, Malawi; eumar@medcol.mw; 7 Centre for Public Health, Equity and Human Flourishing, Torrens University Australia, Adelaide, SA 5000, Australia; nelsensius.fauk@torrens.edu.au; 8 Department of Basic Nursing, Faculty of Nursing, Universitas Airlangga, Surabaya 60115, East Java, Indonesia; hidayat.arifin@fkp.unair.ac.id; 9 Research Group in Medical-Surgical Nursing, Faculty of Nursing, Universitas Airlangga, Surabaya 60115, East Java, Indonesia

**Keywords:** coronary heart diseases, self-care, perspective, qualitative study

## Abstract

**Background/Objectives**: Self-care experiences and understanding of coronary heart disease (CHD) play a pivotal role in the management of CHD and can contribute to positive health outcomes. This qualitative study aimed to explore the views and experiences of CHD patients, their families, and Indigenous leaders about self-care practices of CHD. **Methods**: A qualitative design employing semi-structured interviews and a focus group discussion was used. Employing purposive sampling, 49 respondents, comprising 30 patients, 10 family members, and 9 Indigenous leaders, were recruited and interviewed from April to September 2022. The data were analyzed using content analysis. **Results**: Five major categories were constructed: (1) inadequate knowledge and early symptom identification; (2) self-care activities for physical, psychosocial, and spiritual needs; (3) family and kinship support for self-care efforts; (4) barriers to self-care including physical, psychological, and access issues; and (5) health improvement expectations based on culturally sensitive health education. **Conclusions**: The study reveals significant gaps in knowledge about CHD and the identification of early symptoms among patients, families, and Indigenous leaders. Despite efforts to meet physical, psychosocial, and spiritual needs, self-care is hindered by various barriers, including limited access to healthcare and entrenched habits. The support from family and kinship systems is crucial for self-care. Participants expressed a strong desire for culturally tailored health education and better health control to improve heart health outcomes.

## 1. Introduction

The Global Burden of Disease study and the World Health Organization have reported cardiovascular disease (CVD) as the leading cause of death worldwide [1]. Over the decade 2010–2019, the number of deaths attributed to CVD increased from 15.8 million to 18.6 million, according to recent studies [2]. Among CVD subtypes, coronary heart disease (CHD) ranked first, and the CVD-related mortality rate increased from 7.7 million to 9.1 million globally [2]. Indonesia is one of the countries with the highest CVD mortality rate in the world. The number of deaths increased from 527,000 in 2010 to 651,000 in 2019, with CHD deaths rising from 196,000 in 2010 to 245,000 in 2019 [2,3].

CHD is a complex condition influenced by multiple risk factors that contribute to its onset and progression. The key risk factors identified include age, gender, family history, hypertension, dyslipidemia, smoking, diabetes, obesity, physical inactivity, and psychosocial elements [4]. Age is a particularly significant risk factor, as the incidence of CHD rises with advancing age, likely due to the cumulative effects of other risk factors such as hypertension, elevated cholesterol levels, and sedentary behavior, which become more common as individuals age [4,5,6]. Both active and passive smoking greatly elevate the risk of CHD by promoting arterial plaque formation and decreasing oxygen supply to the heart. This strongly links smoking to the development of CHD [7]. Additional risk factors, including hypertension, dyslipidemia, diabetes mellitus, obesity, lack of physical activity, and poor dietary habits, also have strong associations with CHD [4,8,9]. Self-care barriers among patients with coronary heart disease (CHD) in Indonesia include physical and psychological challenges, low adherence to treatment, and medication side effects [10]. Additionally, the duration of illness has been significantly correlated with self-efficacy in CHD patients [11]. Barriers to healthcare access, such as high costs and care that is not well-adapted to the psychosocial needs and contexts of patients, have also been identified as significant factors [12].

Engagement in self-care is therefore essential to building a healthier life and preventing CHD and other types of CVDs [13]. Self-care consists of several components, including maintenance, monitoring, and management, which are altogether crucial in controlling CHD [14]. Implementing self-management techniques can help patients with CHD feel more confident about managing their condition [15]. It has been reported that patients with CHD who received self-management interventions demonstrated positive results in keeping good eating habits, medication adherence, and participating in physical activity, which contributed to better health outcomes and quality of life [15].

In addition to patients with CHD, their families play an important role in the implementation of self-management through emotional, appreciative, instrumental, and informational support, leading to better health outcomes in chronic disease patients such as those with CHD [16,17]. Indigenous leaders are also reported to have a significant role in enhancing self-management. Previous research showed that the cultural background of Indigenous leaders influenced the perceptions of self-management [18], while social support and self-efficacy correlated with the adherence of patients to medications [19,20]. Given that Indonesia is a multicultural country, the perceptions of individuals within families and communities about health issues are also influenced by Indigenous leaders, particularly in West Sumatra, known as the Minangkabau ethnic group [21]. Therefore, involving Indigenous leaders in supporting CHD patients can be a beneficial strategy to improve self-management and medication adherence.

Among the other provinces in Indonesia, West Sumatra ranks tenth in terms of heart disease prevalence. The percentage of heart disease in the province increased from 1.2% in 2013 to 1.6% in 2018, higher than the national average of 1.5%, highlighting a high risk of CHD among its population [22,23]. Available evidence has suggested that lifestyle patterns, including diet and smoking habits, are closely related to the risk of CHD among people in this region [24]. Dietary patterns such as consuming saturated fats aggravate the condition of atherosclerosis and increase the potential risk of CHD [24,25,26].

Despite the aforementioned research, there is a paucity of evidence on the views and experiences of CHD patients, their families, and Indigenous leaders in Indonesia regarding self-care practice or self-management of CHD. In addition, previous studies have mainly focused on assessing the associations of various risk factors and CHD; thus, there is a lack of qualitative inquiry on this topic. Therefore, the study aimed to qualitatively explore in depth the views and experiences of Indonesian CHD patients, their families, and Indigenous leaders about self-care practice or management of CHD.

## 2. Materials and Methods

### 2.1. Design

A descriptive qualitative research design was used to explore the views and experiences related to self-care management of CHD. Qualitative descriptive research aims to provide a comprehensive summarization of specific events experienced by a single individual or group of individuals [27]. The primary rationale for employing the descriptive method was to offer clear explanations of experiences and perceptions [28].

### 2.2. Participants and Setting

This research was carried out in two hospitals in West Sumatra that offer cardiovascular services in outpatient settings. After receiving the chief cardiovascular outpatient nurse’s approval, participant recruitment was carried out. There were three different participant groups recruited using a purposive sampling technique. By using purposive sampling, the researchers ensured that the study included participants who could provide the most valuable insights, thereby enhancing the overall quality and relevance of the research findings. This included patients and their families as well as Indigenous leaders across nine districts based on the region from which the majority of patients originated.

The department head nurses helped with recruitment by examining patients’ medical histories and conditions according to the inclusion criteria. These criteria included (1) patients with CHD according to their medical record, (2) individuals under the age of 70, (3) willing to share life and self-care experiences of CHD, and (4) able to speak Bahasa Indonesia (Indonesian language) and Minang (local language). The patients and families were facilitated in meeting the research team, and then informed consent was obtained for their willingness to participate in an in-person interview. Although 35 patients were eligible to participate, only 30 volunteers were available.

Family members who accompanied patients with CHD to the hospital were recruited as participants. A total of 15 were contacted, but only 10 agreed to take part in this research. Family members had to meet the following requirements to be considered for inclusion: (1) be willing to participate in the interview process, (2) able to speak Bahasa Indonesia (Indonesian language) and Minang (local language), and (3) aged 70 years or below. Face-to-face interviews were conducted with family members who consented to participate.

To recruit Indigenous leaders (n = 9), formal permission was sought from the Minangkabau Nature Customary Density Institute (Lembaga Kerapatan Adat Alam Minangkabau/LKAAM), West Sumatra. This recruitment was based on the region of the patients and families according to the demographic data collected during interviews. Indigenous leaders were required to meet two criteria to be included: (1) they must be open to interviewing, and (2) they must be known by the titles “Datuak” and “Bundo Kanduang”.

### 2.3. Ethical Consideration

The study obtained approval from the Institutional Review Board (IRB) of Universitas Indonesia, confirming adherence to ethical standards. The IRB’s approval signifies a thorough review of potential risks and benefits, ensuring participants’ rights, safety, and well-being. Participants were fully informed of the study’s purpose, objectives, and procedures, and written informed consent was obtained, highlighting voluntary participation. They were assured they could withdraw at any time without repercussions. Confidentiality was rigorously maintained, with interview data and transcripts anonymized. Unique identifiers (e.g., P for patients, F for family members, I for Indigenous leaders) replaced names to protect privacy. No identifiable information was included in the reports or publications. Interviews were conducted privately and comfortably, respecting cultural and personal sensitivities. Interviewers followed culturally sensitive protocols, particularly given the involvement of Indigenous leaders, ensuring ethical considerations were deeply integrated into the research practice.

### 2.4. Data Collection

Data collection was carried out from April to September 2022 using face-to-face, semi-structured interviews with patients and families to gather information about their experiences and perceptions regarding CHD self-care. Follow-up interviews were conducted with seven participants to gather additional information to fill gaps as well as for clarification and confirmation. Individual interviews with patients were performed in the hospital’s outpatient meeting room before or after visiting a doctor for a check-up. Meanwhile, interviews with families were conducted separately per time and place agreed upon, such as at home or the patient’s workplace. The main components of the questions asked were (a) understanding of the disease, (b) self-care that had been carried out, (c) perceived barriers, (d) support in carrying out self-care, and (e) expectations. The time required for the interview was about 30–60 min in both patients and family groups.

Focus group discussions (FGDs) were used to conduct interviews with Indigenous leaders to acquire a comprehensive understanding of the societal concerns surrounding CHD [29]. A total of nine Indigenous leaders were gathered in a room according to the initial agreement. The moderator, who was also the head of the Indigenous leaders, initiated the discussion, followed by the interviewer asking questions related to their perspectives about CHD in the Minangkabau ethnic community. The question component consisted of (1) the understanding and views about CHD as well as the lifestyle of the Minangkabau ethnic community, (2) treatment of the sick based on the culture, (3) cultural system toward the sick, (4) perceived barriers in managing CHD, (5) support, and (6) health expectations. The FGD lasted approximately 60 to 90 min after all participants answered all the questions.

The number of patients and family members included was determined based on the adequacy of the data collected, ensuring a reasonable range of experiences to address the aims of the research. During the interview, the interviewer and participants agreed to be recorded audio-visually, and information was kept confidential. All interviews were recorded and transcribed verbatim. Demographic data including age, gender, education level, marital status, and duration of CHD diagnosis were collected from patients and families using survey instruments. The detailed guidelines for the interview is presented in Table 1.

Individual interviews and FGDs were conducted by the interviewer (AW), who possesses a master’s educational background in cardiovascular nursing and is experienced in conducting qualitative research. Additionally, two nurses assisted in data collection and note-taking during interviews and FGDs. One was a cardiac nurse in a hospital, and the other had an interest in the cardiovascular field. The interviewers (AW, YR, SN), including the moderators, were female and native Minangkabau speakers. Researchers reached saturation in data collection when no new information emerged from participants during interviews. This indicated that the researchers had obtained a comprehensive understanding of the topic or phenomenon being studied, and further interviews or data collection were unlikely to yield new insights or perspectives that would significantly add to this understanding.

The interviewers introduced themselves, explained the purpose of the research, and obtained written informed consent from each participant. Interviews and FGDs were conducted in a private, quiet, and comfortable location. The reporting guidelines followed the Standards for Reporting Qualitative Research (SRQR) [30].

### 2.5. Data Analysis

The verbatim transcripts of all interviews and FGDs were analyzed using Elo and Kynga’s approach, which explains qualitative research based on content analysis (inductive and deductive) to create themes. This research specifically used the inductive approach, as it was considered well suited for exploring essential, complex, and delicate nursing phenomena and content analysis. The content analysis phases consisted of (1) preparing, (2) organizing, and (3) reporting [31]. The interviews, conducted originally in the Minangkabau language, were translated into Indonesian and then to English before being transcribed.

One of the research team members (AW) reviewed the whole transcript during the preparation phase by listening to audiovisual recordings to confirm the accuracy of the transcript. The preparation stage entailed selecting the unit of analysis and comprehending the data as a whole. The unit of analysis focused on understanding words or sentences as individual units of content based on the interviewer’s questions and latent information such as field notes. The goal was to comprehend the data, which required multiple readings. In the organizing phase, an inductive approach was used to analyze the data after achieving a comprehensive understanding.

This technique comprised open coding, category creation, and abstraction throughout the organizing step. The phase began with re-reading the interview results for manual coding, which was derived inductively from the data, while the topic’s inductive code was obtained from the interview rules. The open coding procedure began with a re-reading of all transcripts of the interviews by members of the data analysis team (AW and SS), followed by determining the keywords found. The essential words were coded, and a manual was created, which included code names, meanings, and textual data.

The initial code manual was created at this point, and it was then applied to the remaining transcripts. The coders (AW and SS) met periodically to discuss the coding process, and any issues were fixed by a third member (HA), who re-read the data. The category listings were then organized under higher-level headings following the open coding. Data grouping aimed to decrease the number of categories by combining those that were related or dissimilar into a larger and higher order.

Categories were created to promote comprehension as well as provide knowledge and means of describing the phenomenon. In the process of creating categories using inductive content analysis, the research team decided which items to group based on interpretation. Furthermore, abstraction referred to developing a general description of the research issue, and each category was named using words that described the content. Categories and subcategories were grouped as primary when they had related occurrences or events. In the final phase, the reports and findings of the analysis process were presented in the form of categories or conceptual maps [31].

### 2.6. Trustworthiness

Trustworthiness was assessed using the criteria of credibility, confirmability, dependability, and transferability [32]. (1) Credibility was ensured through the use of member checks and triangulation, which involved gathering data from multiple sources including direct observation, field notes, and medical records. Meanwhile, (2) confirmability was established by sending the interview findings to the participants and then asking for their feedback to obtain approval. An external check method was also employed on four patients with CHD who had the same characteristics but were not involved in the interview. Validating the findings with external CHD patients helped verify if the results resonated with individuals who share similar experiences but were not part of the original study. This process enhances credibility by ensuring that the researchers’ interpretations accurately reflect the experiences of the target population. It also identifies potential gaps by incorporating additional insights from external patients that may not have been captured initially. Moreover, it reduces researcher bias by including viewpoints from individuals outside the original data collection process. Finally, if external patients confirm the findings, it suggests that the results may be applicable to a broader population of CHD patients with similar characteristics. (3) Dependability was maintained by involving an expert in qualitative research to audit and analyze the different processes. Additionally, (4) transferability was achieved by summarizing and then making a narrative explanation of the interview results.

## 3. Results

### 3.1. Characteristics of Participants

The research comprised 30 patients with CHD, 10 family members, and 9 Indigenous leaders as participants. Demographic characteristics, such as gender, age, education level, marital status, and length of CHD diagnosis, are summarized in Table 2. The mean age of participants was 57.10 ± 5.81 years (40 to 69), and most (76.7%) were male. Furthermore, the highest level of education was senior high school (60%), and 26 (86.7%) were married. The average length of time after CHD diagnosis was 3.2 ± 2.2 months (1 to 10), while the mean age of family participants was 48.10 ± 6.1 years (36 to 56). All family participants were wives of patients, and six (60%) had a college education background. Indigenous leaders had a mean age of 53.33 ± 9.23 years (33 to 61), eight of them (88.9%) were male, and nine of them graduated from college (100%).

**Figure 1:**
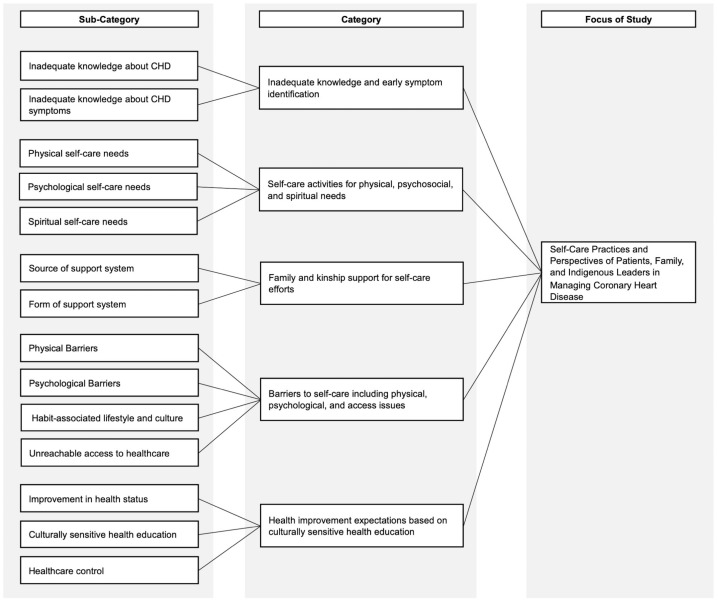
Category distribution of self-care practices and perspectives in managing coronary heart disease patients.

The findings were grouped into five major categories: (i) inadequate knowledge of CHD and identification of early symptoms during heart disease; (ii) activities carried out by participants to fulfill physical, psychosocial, and spiritual self-care needs; (iii) family and kinship systems as a source of support and experience in supporting self-care; (iv) perceived barriers to self-care such as physical and psychological barriers, habits, and unreachable access to healthcare; and (v) expectation of improvement in heart health status with health education based on Minangkabau culture and health control. The results revealed 40 units of analysis, 14 subcategories, and 5 categories as shown in Figure 1.

### 3.2. Category Distributions

#### 3.2.1. Inadequate Knowledge and Early Symptom Identification

The results indicated that all three groups expressed a lack of knowledge about CHD and its related symptoms. Several misunderstandings were found in the participants’ responses, as some were aware only of CHD causes, while others associated it with chest pain. Participants were unaware of their CHD because they had not experienced any symptoms. A 57-year-old man recalled, “*I didn’t know that I had heart disease*” (P12). This response was further reinforced by statements in the family group, wherein seven out of ten did not understand CHD. According to a 56-year-old wife, “*I didn’t know that my husband had heart problem, two days ago he complained of chest pain then recovered and the next day he felt chest pain again*” (F1). Among Indigenous leaders, participants had some understanding that CHD was related to diabetes, hypertension, and cholesterol, but their knowledge was limited. As expressed by a 55-year-old man, “*I don’t know CHD but the main coronary causes are diabetes, cholesterol, and hypertension*” (I2).

Regarding the lack of knowledge about identifying CHD symptoms, the participants were unable to recognize the early signs and often confused them with heartburn, colds, excessive sweating, nausea, and vomiting. These symptoms were commonly referred to as “sitting wind”. In the patient group, a 54-year-old man stated, “*I assumed maybe this is a cold or maybe it’s an ulcer, accompanied by nausea and it didn’t feel like I was having heart attack*” (P1). Furthermore, family members did not recognize the initial symptoms experienced by patients as signs of a heart attack. A 53-year-old female family member recalled, “*So, we thought it was still a stomach ulcer, when he was vomiting at night, and he was in a cold sweat*” (F2). Among Indigenous leaders, participants recognized the early symptoms of CHD, such as angina or other signs of heart disease. However, their understanding of the signs was unclear, as expressed by a 55-year-old male participant, “*CHD has long been associated with the term ‘sitting wind’ and disease suddenly arrived*“ (I2).

#### 3.2.2. Self-Care Activities for Physical, Psychosocial, and Spiritual Needs

The activities were carried out by the patient group, family members, and Indigenous leaders, who provided physical, psychosocial, and spiritual support. In the patient group, participants emphasized the need for physical self-care, including regular rest and sleep, as stated by a 57-year-old man, “*I also manage rest and sleep*” (P10). Patients also gradually manage physical activities, starting with light work, and often needing the assistance of others. A 53-year-old male participant who demonstrated hand movement exercises as a form of light physical activity stated, “*I did light activities first because I had a ring on my heart that had just been placed*” (P7). Furthermore, family members assisted patients in managing their activities, as explained by a 55-year-old wife, “*I’m the one who stopped again, so after installing the ring, it can no longer be reduced to the activities to the rice fields, and if you want to go to the rice fields, just control it*” (F8). Self-management by limiting and reducing high-risk meals assisted in maintaining nutritional needs, as stated by a 53-year-old man, “*eating offal is not allowed and restricted, just eat solid meal*” (P9). Family also helped patients in meeting nutritional needs by preparing food, as expressed by a 54-year-old wife, “*Then in terms of food, I always take care of him by making sautéed food with very little oil and minimal salt*” (F4). Furthermore, smoking management as a means to fulfill physical needs was specifically mentioned in the patients group. A 59-year-old man stated, “*since I had a heart attack, I have not smoked*” (P5). All patients group participants acknowledged that they kept other physical needs by complying with the treatment and taking routine medication. This was explained by a 57-year-old man as follows, “*Monthly control from the hospital to get routine medication*” (P20). In the family group, participants mentioned their involvement in meeting patients’ physical needs by preparing medications, as stated by a 53-year-old wife, “*Prepare his medicine and always put it in a briefcase*” (F2). Meanwhile, in the Indigenous leader group, seven participants revealed that the Minangkabau people fulfill their self-care needs, especially physically, by utilizing herbal plants as medicine. These herbs can prevent the formation of cholesterol in the blood, as described by a 46-year-old man, “*Some of these foods cause CHD, and there are also antidotes using herbs*” (I5).

The practice of fulfilling psychological self-care needs in the patient group was demonstrated by emotional management such as calming the mind, dampening emotions, and avoidance. This was expressed by participants in the patient group, with a 57-year-old man stating, “*If we feel afraid of this disease then we just calm our minds*” (P12). Silence was also employed by family members to help control patients’ emotions, as revealed, “*The trigger for his anger was there, he felt we were always wrong, and I just kept quiet, not responding to his anger*” (F3). Participants in the patient group also engaged in relaxation techniques and deep breathing to address psychological needs. According to a 57-year-old man, while demonstrating deep breathing, “*I catch my breath slowly*” (P20). In the family group, meeting psychological needs involved providing comfort and support, as expressed by six participants. A 36-year-old woman stated that while taking deep breaths, “*Even with the many problems between me and my husband, I teach him how to relax and take a deep breath*” (F5). Moreover, participants revealed self-motivation as a key factor in meeting psychological needs. This was expressed by a 54-year-old man, *“I motivate myself to be enthusiastic about trying to heal*” (P27).

Patients also performed self-care spiritually by listening to Murottal Al-Qur’an, as expressed by a 53-year-old man, “*The way I calm my mind is to listen to the murottal Al-Qur’an and religious advice on YouTube*” (P6). Participants admitted to reciting istighfar (Dua for forgiveness) and praying, as stated by a 48-year-old man, “*I recite istighfar only, and astaghfirullahaladzim when I feel angry*” (P18). Family also assisted in meeting patients’ spiritual needs by accompanying them to prayers at the mosque, as stated by a 47-year-old woman, “*We go to the mosque and recite prayers together*” (F4). According to all Indigenous leaders, meeting self-care needs through prayers and spiritual activities is deeply rooted in the Minangkabau culture. As stated by a 57-year-old man, “*I am sure that all Minangkabau ethnicity, if they are sick, always draw closer to Allah SWT with patience and prayer*” (I1).

#### 3.2.3. Family and Kinship Support for Self-Care Efforts

Support systems for patients during illness stemmed from the nuclear family. A woman aged 62 years stated that, “*I get support from my husband for healing*” (P14). The extended family was identified by participants as a source of support. This was stated by a 60-year-old man, “*Support from extended family was also there*” (P21). All of the family interviewed admitted that they received support from other relatives, as expressed by a 48-year-old woman, ”*There was support from all parties, especially our extended family, ranging from taking him to the hospital to when he was referred*” (F7). As expressed by an Indigenous leader, the tribal kinship system played a role in providing social support to patients. According to a 33-year-old man, “*In emergencies, the Minangkabau culture dictates that all decisions regarding medical expenses are prioritized, and if there are none, deliberations about using heirloom property are held*” (I8).

One form of support that patients valued was attention and reminders, as expressed by a 48-year-old man, stating, “*My children always reminded me not to smoke*” (P18). Another support according to the statements of participants was preparing medicine and food as well as accompanying them to treatment and providing medical assistance funds. A 57-year-old man, while pointing to a shirt pocket filled with money, stated that, “*Helping with medical expenses by donating*” (P20). Family provided comprehensive support, such as meeting patients’ needs. This was expressed by all family participants, including a 46-year-old wife, stating, “*I always take care of meeting his nutritional needs by making pan-fried food with very little oil and salt*” (F4).

#### 3.2.4. Barriers to Self-Care, Including Physical, Psychological, and Access Issues

The barriers encountered when practicing self-care included physical and psychological, as well as smoking habits, difficulties in accessing health facilities, and the lack of health information in the community. In the patient group, the perceived barriers reported by 18 participants were shortness of breath, for example, “*Only so far until now breathing is still heavy and if the activity is fast, it leads to a shortness of breath*” (P8). Chest pain and palpitations were also mentioned by participants. A 61-year-old man stated, “*If this chest hurts overly (holding his chest), there is a sense of burden*” (P16). Additionally, lack of appetite and sleep disturbances were felt by participants, as expressed by a 58-year-old woman stating, “*Sleeping in supine position causes shortness of breath, and it makes me feel insomnia*” (P22).

The results also showed that psychological barriers were felt in patients and family groups. The psychological complaints included frequent emotions, anxiety, and fear, as stated by a 60-year-old man, “*I’m anxious and afraid. People say that if they have a heart attack, they can die quickly because my brother and nephew died suddenly*” (P21). Psychologically, the barrier encountered by family members in treating patients was the difficulty in maintaining their lifestyle. This was revealed by three family participants, including a 47-year-old wife, stating, “*Currently, regarding the food that will be prepared for my husband, I am free to eat high fat because if high-fat food is prohibited, my husband is stressed*” (F4).

Based on the results, barriers in the form of habits were also felt by both patients and family groups. Patients’ pre-existing habits, such as consuming traditional food and smoking, as well as the influence of the work environment, served as obstacles to maintaining self-care. This was expressed by a 63-year-old woman who stated that, “*This traditional Minangkabau food is actually healthy, but excessive preparation methods increase the fat content, and because it has become a habit, it is difficult to change the food to just boiled food*” (P13). In addition, participants felt they had lost their role in cultural practices. According to a 57-year-old man, “*Participating in traditional events (Baralek) presents a challenge because the treats offered are difficult to resist, and not being present at these activities as a native of Minangkabau makes me feel I’m neglecting my role, also, there is a habit of eating together at work”* (P11). The barrier perceived by family was controlling the patient’s habits, as expressed by a 36-year-old wife, “*My husband found it difficult to control eating and other activities*”, “*My husband is difficult to control regarding eating and other activities, so it is challenging to change to his healthy lifestyle*” (F5). Family also mentioned cultural factors, as commented by a 42-year-old woman, “*This area is famous for Minangkabau cuisine, so I also don’t control my husband’s food*” (F7).

Barriers related to inaccessible healthcare were reported, as expressed by a 54-year-old man, “*Health facilities were far from where we live and we do not have enough money for transportation*” (P1). In the Indigenous leader group, participants revealed that the perceived barrier to self-care was the lack of socialization about CHD. According to a 49-year-old man, “*There was lack of socialization in the community despite the high risk of CHD*” (T7, 49 years, male).

#### 3.2.5. Health Improvement Expectations Based on Culturally Sensitive Health Education

The results showed that expectations were expressed by the three groups, namely patients, family, and Indigenous leaders. The hope primarily revolved around improving health status, such as preventing repeated attacks, increasing health education by involving family and Indigenous leaders as socio-cultural support, as well as ensuring proper patient care at home under the guidance of health professionals. In the patient group, participants hoped for an increase in health status, such as recovery and absence of re-attacks, as expressed by a 57-year-old male patient, “*I hope that there will be no recurrence of heart disease, no readmissions, and to stay healthy*” (P10). Families of patients also expressed their hope for preventing recurrent heart attacks. According to a 53-year-old woman, “*Hopefully my husband will stay healthy by managing his lifestyle so that there will be no more heart attacks*” (F2).

In terms of increasing health education, socio-cultural support was expressed by participant groups. In the patient group, participants expressed their hope for health education involving the community in Minangkabau, including a 58-year-old man stating, “*There should be counselling to the Minangkabau community*” (P4). Furthermore, patients expected health education to involve Indigenous leaders, as stated by a 57-year-old man, “*Invite indigenous leaders in this counselling*” (P12). In the family group, participants expressed their hope for health education when caring for or accompanying patients to the hospital. This was reported by a 48-year-old woman, stating, “*We really need health education in treating patients at home*” (F9). Indigenous leaders also declared their hope for strategies to reduce and prevent CHD through health socialization events. This was revealed by a 61-year-old man, who stated, “*Maybe we need a strategy here to sensitize the Minangkabau community about CHD by involving indigenous leaders*” (T4).

Finally, the participants expressed their hope for post-treatment monitoring at home by health workers. This was expressed by a 57-year-old man who stated that, “*There is monitoring from health workers after being at home*” (P10). Two families highlighted the need for structured daily monitoring of patients. According to a 49-year-old woman, “*We hope that there will be monitoring of patients and education on daily activities involved in caring for patients*” (F10).

## 4. Discussion

The study’s findings highlight a critical issue in the management of coronary heart disease (CHD) among the Minangkabau ethnic group in West Sumatra, Indonesia. The research revealed that patients, their families, and even Indigenous leaders demonstrated inadequate knowledge about CHD and struggled to identify its early symptoms. This lack of understanding was consistent across all three groups, regardless of their education levels. Patients often misinterpreted CHD symptoms, attributing them to less serious conditions like gastritis or colds. This knowledge gap can lead to delayed treatment, which is crucial for CHD management and patient survival. The study emphasizes the need for targeted health education initiatives that are culturally appropriate and involve all stakeholders, including Indigenous leaders. Improving the awareness and understanding of CHD symptoms and risk factors could potentially lead to earlier detection, better self-care practices, and improved health outcomes in this population.

In category one, the results were consistent across all three groups, indicating inadequate knowledge about CHD and the early symptoms of heart attack. Previous research conducted in Malaysia showed that a poor understanding of CHD was related to age, gender, and level of education [33]. Similarly, the data revealed that approximately 75% of patients’ family members had limited knowledge about CHD [34]. It was found that the level of education and experience influenced their ability to recognize heart disease along with the signs and symptoms [35].

The demographic data obtained showed that the highest education level of most patient participants was high school. Although the education level of the family and Indigenous leader groups was significant, they had limited information about CHD, resulting in poor knowledge. In the general population, CHD is commonly associated with sudden stabbing chest pain, but more than 80% do not recognize atypical symptoms such as shoulder pain, shortness of breath, nausea, and pain. Consequently, patients often attribute their pain to gastritis and colds [36]. Understanding the symptoms and warning signs is important as it affects the management of CHD and patients’ survival [37]. According to previous research, only a small percentage of people identified chest pain as a heart attack, leading to delayed treatment [38].

All three groups of participants interviewed in this research reported engaging in self-care efforts. The actions of patients and family groups were influenced by the knowledge of CHD, the emotional response, and the confidence to practice self-care [39,40]. High motivation plays an important role in complying with self-care practices [41]. According to [42], CHD patients who incorporated spiritual self-care experienced positive effects on their life expectancy. These patients thank God, accept the pain, and consider heart attack a life-or-death warning [43]. This research found similar results, where patients adopted spirituality to cope with their pain by always remembering God. In the Minangkabau culture, it is believed that spiritual improvement is a necessary aspect of self-care when one is sick. Additionally, the community relies on herbal ingredients for disease prevention and treatment, and as seasonings in traditional dishes (ethnobotanical and ethnomedicine). According to research in several areas of West Sumatera, there are approximately 200 species of medicinal plants and about 100 species for food [44,45].

Four barriers that patients face after acute coronary syndrome, namely, psychological, physical, lack of knowledge, and non-compliance [46]. The perceived barriers after percutaneous coronary intervention in patients with CHD are physical and psychological barriers, non-adherence, and side effects of medications. Another study on barriers after CHD reported difficulty in quitting smoking due to addiction, as well as challenges with physical activity and adhering to a specific diet [47]. Patients experienced increased difficulties with physical activities (kinesiophobia) during cardiac rehabilitation, with greater psychological barriers than biological factors [48]. CHD is one of the leading causes of limited activity and participation in activities [49]. Psychiatric morbidity, such as anxiety and depression, occurred frequently in patients, and it was found that 42.4% experienced anxiety at high levels [50]. The barriers experienced by patients in changing lifestyle habits included carrying out physical activity, changing dietary habits, and quitting smoking. These barriers are influenced by sociodemographic and health-related patient characteristics [51]. Behavior, environment, and culture are social determinants contributing to cardiovascular disease, accounting for up to 80% of barriers faced by patients in self-care [52]. Ethnocultural practices can also present barriers to patients’ cardiac healthcare [53]. Overall, there is a lack of knowledge about CHD and its management in the general public, which acts as a barrier to controlling disease [54].

The support perceived by patients and provided by family along with Indigenous leaders has also been reported in qualitative research. Family-provided self-care can improve the subjective well-being of CHD patients [55]. Engaging family members in the treatment improved patients’ and family outcomes, increased satisfaction, improved achievement of medical goals, as well as facilitated the recovery and psychological well-being of patients and families [56]. Furthermore, social support from family and friends has been linked to lower acceptance rates of returns and better adherence to treatment [57]. Culture also affects the conduct of self-care, especially in relation to maintenance behaviors, with kinship ties playing an important role in medication adherence [58]. Social support from different people, including spouses, family, peers, and kinship, can increase self-efficacy in medicine and physical activity [59]. The Minangkabau community is one of the tribes in Indonesia that adheres to the matrilineal kinship system formed from the maternal (female) lineage and functions as a support system in all situations [60].

Hopes for self-care were expressed by the three groups, including the involvement of Indigenous leaders based on the Minangkabau customary philosophy. Continuity of care programs implemented by nurses on CHD patients played a crucial role in improving disease monitoring and control and enhancing the quality of life, mental health, and self-efficacy, leading to reduced clinical parameters of blood pressure and sugar [61]. Furthermore, comprehensive nursing education interventions have been shown to effectively improve patients’ psychological state, self-care ability, and quality of life in CHD [62]. Good health literacy is vital for the optimal implementation of self-care and improving the quality of health [63]. Heart health interventions that adopt a culture-centered approach, including the dissemination of health, economic, social, and educational information, are considered feasible for promoting the equitable distribution of resources [64]. Moreover, nursing interventions based on the Minangkabau traditional philosophy, known as “Adat Basandi Syarak Syarak Basandi Kitabullah” (ABS-SBK), is a value system that guides the interaction between individuals and their environment through Islamic values and customs. Implementation of this philosophy ensures that patients receive self-care that aligns with halalan thoyibah (permissible and good) principles [65].

### Strengths and Limitations

The strength of this research lies in its comprehensive inclusion of patients, their families, and Indigenous leaders, providing insights into self-care practices among coronary heart disease (CHD) patients within the Minangkabau cultural context. Data were gathered from diverse spiritual and cultural biopsychosocial aspects, enhancing the depth of understanding. The findings can inform the development of culturally grounded nursing interventions for CHD patients in the Minangkabau ethnic group and similar populations. One limitation was the use of purposive sampling, which may limit generalizability. Additionally, the simultaneous translation of audio recordings posed challenges, though quality checks and retention of culturally significant phrases in the Minangkabau language mitigated potential loss of meaning. Another limitation included overrepresentation of male and deceased family participants, addressed through focused interview questions exploring gender differences in self-care practices.

## 5. Conclusions

In conclusion, this research found five categories related to self-care in CHD explored from the perspectives of patients, family members, and Indigenous leaders. The categories predominantly revolved around the involvement of patients and families with support from Indigenous leaders, in line with the principles of Minangkabau culture. The results are expected to form the basis for developing a holistic and sustainable Minangkabau culture-based nursing intervention model. Future research should refine culturally adapted nursing interventions, assess their long-term sustainability, and explore the specific roles of Indigenous leaders in supporting self-care. Integrative studies combining cultural and biomedical approaches could optimize CHD management strategies globally.

## Figures and Tables

**Table 1:** Interview guidelines.

Participant Group	Questions
Patients	Understanding and Views about CHD
	*What do you know about coronary heart disease (CHD)?*
	Treatment of the Sick Based on the Culture
	*How was your experience when you had a heart attack until now (after CHD)?*
	Self-Care
	*How do you do self-care?*
	Support
	*What support do you get when doing self-care?*
	Perceived Barriers in Managing CHD
	*What barriers do you experience when doing self-care?*
	Health Expectations
	*What are your hopes for self-care?*
Families	Understanding and Views about CHD
	*What does the family understand about CHD?*
	Treatment of the Sick Based on the Culture
	*How is the family’s experience when dealing with heart attack patients until now?*
	Cultural System Toward the Sick
	*What is the family’s experience in caring for patients with CHD?*
	Support
	*What support does the family provide when caring for CHD patients?*
	Perceived Barriers in Managing CHD
	*What obstacles do families experience when caring for CHD patients?*
	Health Expectations
	*What are the expectations of the family in caring for CHD patients?*
Indigenous Leaders	Understanding and Views about CHD
	*What does the family understand about CHD?*
	Treatment of the Sick Based on the Culture
	*How is the family’s experience when dealing with heart attack patients until now?*
	Cultural System Toward the Sick
	*What is the family’s experience in caring for patients with CHD?*
	Support
	*What support does the family provide when caring for CHD patients?*
	Perceived Barriers in Managing CHD
	*What obstacles do families experience when caring for CHD patients?*
	Health Expectations
	*What are the expectations of the family in caring for CHD patients?*

**Table 2:** Participants’ Characteristics.

Characteristics	Patients n (%)	Family n (%)	Traditional Leaders n (%)
Gender			
Men	23 (76.7)	0 (0)	8 (88.9)
Women	7 (23.3)	10 (100)	1 (1.1)
Age, *mean ± SD*	57.10 ± 5.81	48.10 ± 6.1	53.33 ± 9.23
Educational Level			
Junior High School	2 (6.7)	0 (0)	0 (0)
Senior High School	18 (60)	4 (40)	0 (0)
Higher Education	10 (3.3)	6 (60)	9 (100)
Marital Status			
Married	26 (86.7)	10 (100)	9 (100)
Divorced	4 (13.3)	0 (0)	0 (0)
Years since coronary heart disease, *mean ± SD*	3.2 ± 2.2		

Abbreviations: n, number; SD, standard deviation.

## Data Availability

The data are available from the corresponding author by reasonable request.

## Public Involvement Statement

No public involvement in any aspect of this research.

## Guidelines and Standards Statement

The reporting guidelines followed the Standards for Reporting Qualitative Research (SRQR).
